# Selective neuronal staining in tardigrades and onychophorans provides insights into the evolution of segmental ganglia in panarthropods

**DOI:** 10.1186/1471-2148-13-230

**Published:** 2013-10-24

**Authors:** Georg Mayer, Christine Martin, Jan Rüdiger, Susann Kauschke, Paul A Stevenson, Izabela Poprawa, Karin Hohberg, Ralph O Schill, Hans-Joachim Pflüger, Martin Schlegel

**Affiliations:** 1 Animal Evolution and Development, Institute of Biology, University of Leipzig, Talstraße 33, D-04103 Leipzig, Germany; 2 Physiology of Animals and Behavior, Institute of Biology, University of Leipzig, Talstraße 33,D-04103 Leipzig, Germany; 3 Department of Animal Histology and Embryology, University of Silesia, Bankowa 9, 40-007 Katowice, Poland; 4 Senckenberg Museum of Natural History Görlitz, Am Museum 1, D-02826 Görlitz, Germany; 5 Biological Institute, Zoology, University of Stuttgart, Pfaffenwaldring 57, D-70569 Stuttgart, Germany; 6 Neurobiology, Institute of Biology, Freie Universität Berlin, Königin-Luise-Str. 28-30, D-14195 Berlin, Germany; 7 Molecular Evolution & Animal Systematics, Institute of Biology, University of Leipzig, Talstraße 33, D-04103 Leipzig, Germany; 8 German Centre for Integrative Biodiversity Research (iDiv), Halle-Jena-Leipzig, Deutscher Platz 5e, D-04103 Leipzig, Germany

**Keywords:** Arthropoda, Ecdysozoa, Nervous system, Onychophora, Panarthropoda, Segmental ganglia, Synapomorphy, Tardigrada

## Abstract

**Background:**

Although molecular analyses have contributed to a better resolution of the animal tree of life, the phylogenetic position of tardigrades (water bears) is still controversial, as they have been united alternatively with nematodes, arthropods, onychophorans (velvet worms), or onychophorans plus arthropods. Depending on the hypothesis favoured, segmental ganglia in tardigrades and arthropods might either have evolved independently, or they might well be homologous, suggesting that they were either lost in onychophorans or are a synapomorphy of tardigrades and arthropods. To evaluate these alternatives, we analysed the organisation of the nervous system in three tardigrade species using antisera directed against tyrosinated and acetylated tubulin, the amine transmitter serotonin, and the invertebrate neuropeptides FMRFamide, allatostatin and perisulfakinin. In addition, we performed retrograde staining of nerves in the onychophoran *Euperipatoides rowelli* in order to compare the serial locations of motor neurons within the nervous system relative to the appendages they serve in arthropods, tardigrades and onychophorans.

**Results:**

Contrary to a previous report from a *Macrobiotus* species, our immunocytochemical and electron microscopic data revealed contralateral fibres and bundles of neurites in each trunk ganglion of three tardigrade species, including *Macrobiotus* cf. *harmsworthi*, *Paramacrobiotus richtersi* and *Hypsibius dujardini*. Moreover, we identified additional, extra-ganglionic commissures in the interpedal regions bridging the paired longitudinal connectives. Within the ganglia we found serially repeated sets of serotonin- and RFamid-like immunoreactive neurons. Furthermore, our data show that the trunk ganglia of tardigrades, which include the somata of motor neurons, are shifted anteriorly with respect to each corresponding leg pair, whereas no such shift is evident in the arrangement of motor neurons in the onychophoran nerve cords.

**Conclusions:**

Taken together, these data reveal three major correspondences between the segmental ganglia of tardigrades and arthropods, including (i) contralateral projections and commissures in each ganglion, (ii) segmentally repeated sets of immunoreactive neurons, and (iii) an anteriorly shifted (parasegmental) position of ganglia. These correspondences support the homology of segmental ganglia in tardigrades and arthropods, suggesting that these structures were either lost in Onychophora or, alternatively, evolved in the tardigrade/arthropod lineage.

## Background

**Figure 1:**
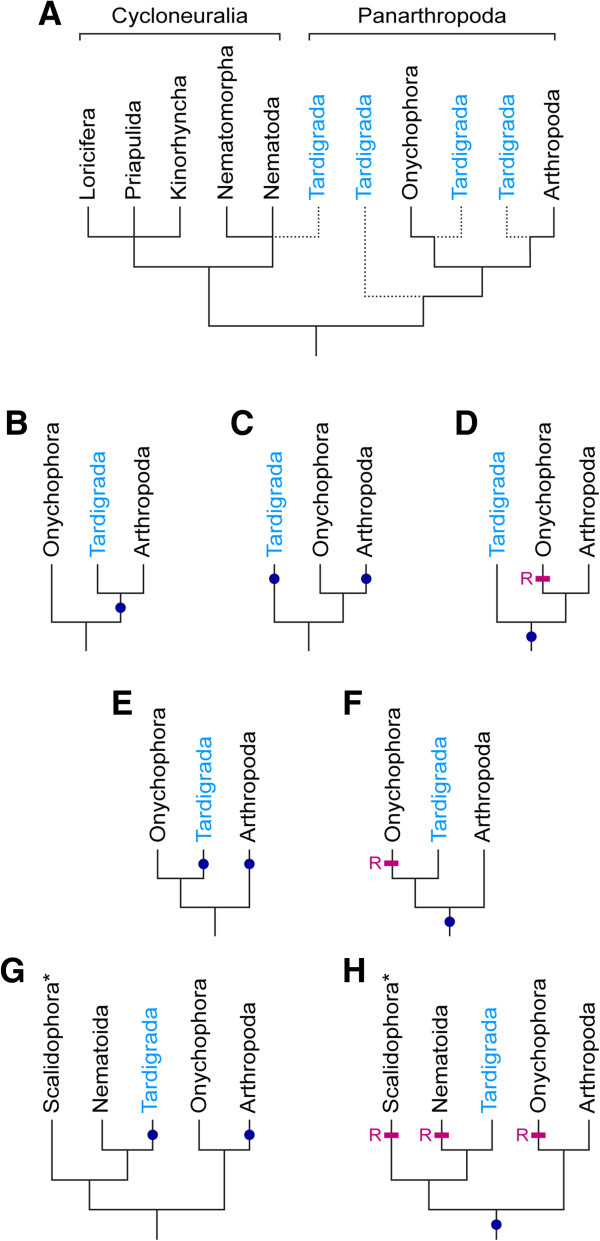
**Alternative hypotheses on the phylogenetic position of the Tardigrada within the Ecdysozoa and the evolution of segmental ganglia. (A)** Tree illustrating controversial views on the phylogenetic position of tardigrades as either the sister group of arthropods, onychophorans, onychophorans plus arthropods, or one of the cycloneuralian taxa (modified from ref. [8]). **(B–H)** Alternative scenarios on the evolution of segmental ganglia (dark-blue dots) in panarthropods, depending on the phylogenetic position of tardigrades favoured. Segmental ganglia might be either homologous structures **(B, D, F, H)** or they might have evolved independently in tardigrades and arthropods **(C, E, G)**. The scenario in **B** is most parsimonious, whereas those in **D**, **F** and **H** require additional losses (magenta bars with an “R”) in onychophorans and/or other ecdysozoans. Asterisks in **G** and **H** indicate that a ventral chain of ganglia-like thickenings also occurs in kinorhynchs, although their homology with segmental ganglia of tardigrades and arthropods is uncertain (see text for further details).

The phylogenetic position of tardigrades is controversial, as they are typically regarded as either the sister group of arthropods, onychophorans, onychophorans plus arthropods, or one of the cycloneuralian taxa, such as nematodes [1-16]. Incongruent topologies based on molecular datasets suggest that Tardigrada is a rogue taxon, the phylogenetic position of which is uncertain (Figure 1A). The problem with molecular phylogenetic approaches might be due to long-branch attraction artefacts, which have been shown to either directly or indirectly affect the inferred topologies [17-19]. These artefacts might have caused the conflicting hypotheses on the position of Tardigrada in different molecular phylogenetic studies [2,4,11-13]. Hence, to rely entirely on molecular datasets may be insufficient when trying to resolve the phylogenetic position of tardigrades within the Ecdysozoa.

An alternative approach to discern the phylogenetic relationships between different animal groups is the comparison of their anatomy, including the organisation of their nervous systems [20,21]. One of the most prominent features of the tardigrade nervous system is the presence of segmentally repeated ganglia in their ventral nervous system [22-25]. The ganglia are accumulations of neuronal cell bodies, which are linked by somata-free connectives along the body [21]. Among representatives of Panarthropoda (Onychophora + Tardigrada + Arthropoda), segmental ganglia occur only in arthropods and tardigrades, whereas somata-free connectives and segmental ganglia are lacking in onychophorans, which instead show a medullary organisation of their ventral nerve cords [8,26-28]. Thus, the question arises of whether segmental ganglia evolved convergently in tardigrades and arthropods, or whether they are homologous structures (Figure 1B–H). If they are indeed homologous, they might either have been present in the last common ancestor of Panarthropoda or even Ecdysozoa (albeit reduced in Onychophora [12]), or they might represent a synapomorphy uniting the tardigrades and arthropods [8].

To clarify this issue and shed light on the evolution of segmental ganglia, we have chosen two approaches. On the one hand, we searched for specific correspondences between the tardigrade and arthropod ganglia by analysing the staining patterns of a variety of established neuronal markers, including antisera raised against tyrosinated and acetylated α-tubulin (a component of neurotubules), the biogenic amine serotonin and the neuropeptides RFamide, allatostatin and perisulfakinin. On the other hand, we performed retrograde dye tracing of leg nerves in the onychophoran *Euperipatoides rowelli* to localise the cell bodies of motor neurons supplying the legs. This approach was necessary to allow a comparison with tardigrades and arthropods because segmental ganglia are lacking in onychophorans and the leg nerves are the most prominent segmental structures associated with nerve cords in these animals [8,26-28].

## Results

### Organisation of the central and peripheral nervous system in the three tardigrade species studied

**Figure 2:**
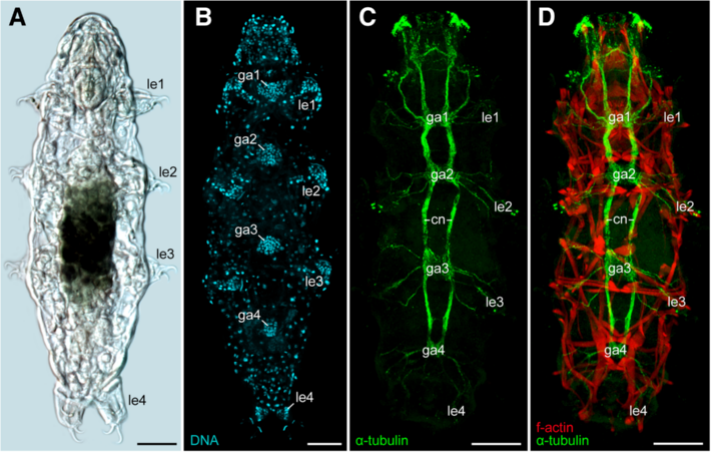
**General anatomy and organisation of the ventral nervous system and position of trunk ganglia in tardigrades. (A)** Light micrograph of a specimen of *Hypsibius dujardini* in dorsal view. **(B–D)** Confocal micrographs of *Macrobiotus* cf. *harmsworthi* specimens (ventral view, anterior is up). Note the anteriorly shifted position of the four trunk ganglia with respect to each leg pair. **(B)** DNA labelling of cell nuclei (SYBR® Green). **(C)** Combined anti-tyrosinated and anti-acetylated α-tubulin immunolabelling to reveal nerve tracts. **(D)** Combined anti-tyrosinated and anti-acetylated α-tubulin immunolabelling (green) and phalloidin-rhodamine staining (red) to reveal the musculature **(**same specimen as in **C)**. Abbreviations: cn, somata-free connectives; ga1–ga4, trunk ganglia 1 to 4; le1–le4, walking legs 1 to 4. Scale bars: 25 μm **(A–D)**.

**Figure 3:**
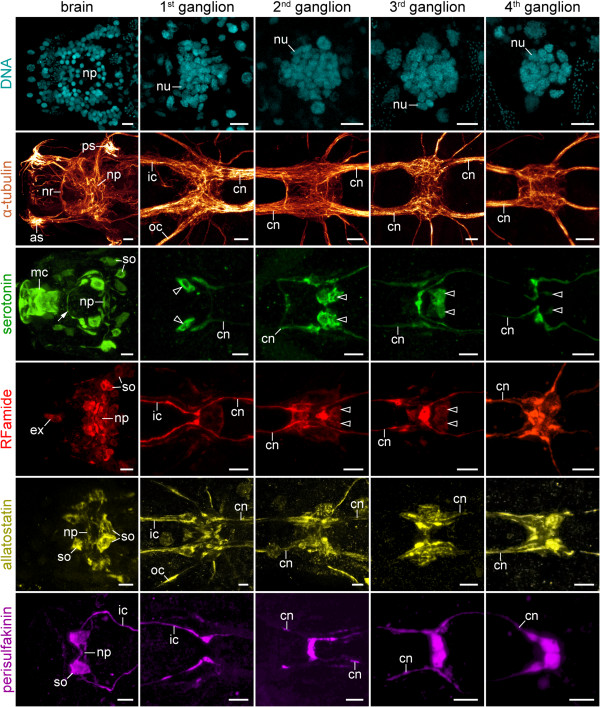
**Organisation of the brain and the four trunk ganglia in tardigrades, as revealed by different markers.** DNA labelling (SYBR® Green), anti-tyrosinated/anti-acetylated α-tubulin immunolabelling, and anti-serotonin-like and anti-RFamide-like immunoreactivity in *Macrobiotus* cf. *harmsworthi*. Anti-allatostatin-like immunoreactivity in *Paramacrobiotus richtersi.* Anti-perisulfakinin-like immunoreactivity in *Hypsibius dujardini.* Confocal micrographs of brains in dorsal view and trunk ganglia in ventral view; anterior is left in all images. Note the contralateral projections and commissure-like structures as well as serially repeated neuronal somata within each trunk ganglion (arrowheads). Note also distinct bilateral symmetry in the arrangement of neuronal somata within the brain as well as in each trunk ganglion. Arrow points to a serotonin-like immunoreactive fibre in the dorsal portion of the buccal ring nerve (see ref. [24] for further details). Abbreviations: as, anterolateral sensory field; cn, connective; ex, extra-cerebral neuronal cell body; ic, inner connective; mc, mouth cone; np, central brain neuropil; nr, buccal nerve ring; nu, nuclei of neurons; oc, outer connective; ps, posterolateral sensory field; so, neuronal somata. Scale bars: 5 μm.

**Figure 4:**
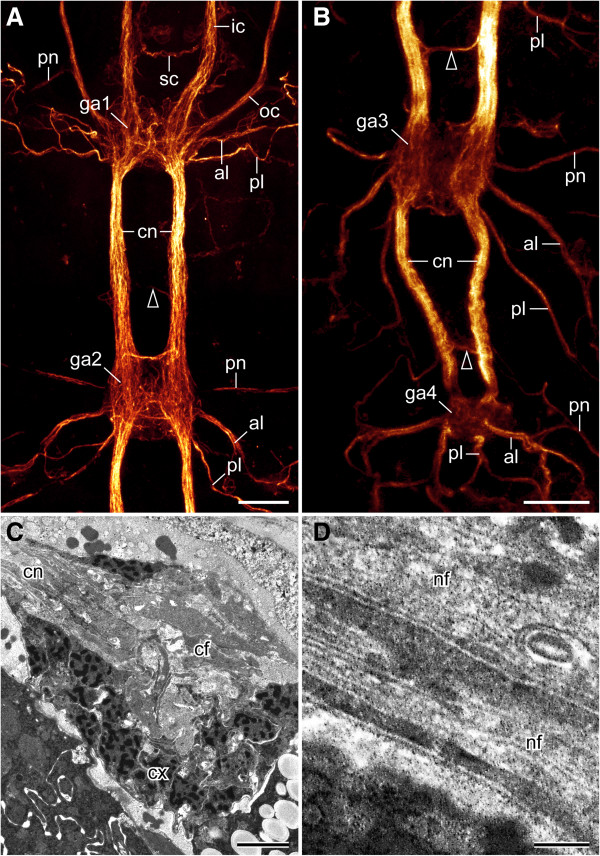
**Organisation of the tardigrade ventral nervous system.** Note the extra-ganglionic, interpedal commissures in front of the second, third and fourth trunk ganglia **(**arrowheads in **A** and **B)**. Combined anti-tyrosinated and anti-acetylated α-tubulin immunolabelling, confocal micrographs **(A, B)** and transmission electron micrographs **(C, D)** from *Macrobiotus* cf. *harmsworthi*. **(A)** Detail of the anterior portion of the ventral nervous system. Anterior is up. **(B)** Detail of the posterior portion of the ventral nervous system. Anterior is up. **(C)** Oblique sagittal section of a trunk ganglion showing the central fibre mass. Anterior is in the upper left corner. **(D)** Transverse section of an interpedal commissure. Abbreviations: al, anterior leg nerve; cf, central fibre mass; cn, connective; cx, cortex; ga1–ga4, trunk ganglia 1 to 4; ic, inner connective; nf, nerve fibre; oc, outer connective; pl, posterior leg nerve; pn, peripheral nerve; sc, commissure of the stomodeal complex. Scale bars: 10 μm **(A, B)**, 2 μm **(C)** and 250 nm **(D)**.

The three tardigrade species studied, including *Macrobiotus* cf. *harmsworthi*, *Paramacrobiotus richtersi* and *Hypsibius dujardini*, show basically the same organisation of the central nervous system, with the brain and the four trunk ganglia being the most prominent features (Figures 2A–D and 3). The brain comprises a dorsal, bilaterally symmetric ganglion, which bears a central neuropil and a defined number of serotonin-like (n ≅ 14), RFamide-like (n ≅ 26), allatostatin-like (n ≅ 12) and perisulfakinin-like (n = 2) immunoreactive somata (see ref. [24] for further details on the architecture of the brain in *Macrobiotus* cf. *harmsworthi*). The brain is linked to the first trunk ganglion via an inner and an outer pair of connectives, whereas the trunk ganglia are joined with each other by only one pair of connectives along the body (Figures 2C, D, 3 and 4A, B). The first three ganglia are similar in size, whereas the fourth ganglion is smaller and contains fewer cells, as revealed by DNA labelling (Figures 2B and 3).

**Figure 5:**
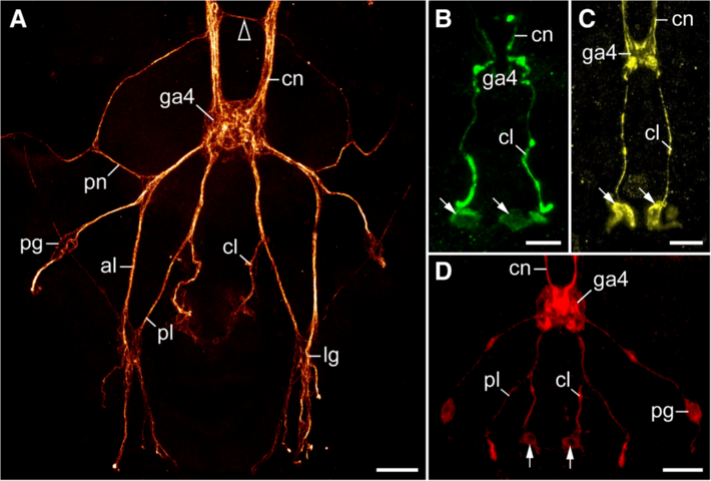
**Organisation of the peripheral nervous system at the posterior end of tardigrades.** Confocal micrographs from *Macrobiotus* cf. *harmsworthi ***(A, B, D)** and *Paramacrobiotus richtersi***(C)**. Anterior is up in all images. **(A)** Combined anti-tyrosinated and anti-acetylated α-tubulin immunolabelling. Arrowhead points to an extra-ganglionic, interpedal commissure in front of the fourth trunk ganglion. **(B)** Anti-serotonin-like immunoreactivity. Arrows point to the two platelet-shaped structures (=putative release sites) associated with cloacal nerves. **(C)** Anti-allatostatin-like immunoreactivity. Arrows point to the two platelet-shaped structures associated with cloacal nerves. **(D)** Anti-RFamide-like immunoreactivity. Arrows point to the somata of immunoreactive neurons associated with cloacal nerves. Abbreviations: al, anterior leg nerve; cl, cloacal nerve; cn, connective; ga4, fourth trunk ganglion; lg, leg ganglion; pg, peripheral ganglion; pl, posterior leg nerve; pn, peripheral nerve. Scale bars: 10 μm **(A, C)** and 5 μm **(B)**.

Each trunk ganglion consists of two hemiganglia that are fused along the ventral midline and show a central fibre mass (Figures 3, 4A–C and 5A). Within this mass, a clear structure of fibre arrangement is not apparent. However, commissure-like bundles of contralateral neurites are present, some of which display serotonin-like, RFamide-like, allatostatin-like and perisulfakinin-like immunoreactivity (Figures 3, 4A, B and 5A–D). In addition to these fibres and bundles of neurites, each trunk ganglion displays a set of serotonin-like and RFamide-like immunoreactive cell bodies that are organised in a bilaterally symmetric pattern and show essentially the same arrangement in different specimens of the same species (arrowheads in Figure 3 and Additional file 1).

Apart from contralateral projections and commissure-like structures within each ganglion, additional extra-ganglionic (=interpedal) commissures occur anterior to the second, third and fourth trunk ganglia (arrowheads in Figures 4A, B, 5A and Additional file 2). These interpedal commissures are not accompanied by neuronal cell bodies, as they are not associated with ganglia but link the connectives, which are somata-free. Each interpedal commissure consists of a bundle of fibres, which in contrast to those in the connectives do not show serotonin-like, RFamide-like, allatostatin-like or perisulfakinin-like immunoreactivity (Figures 4D, 5A and Additional file 3, Additional file 4, Additional file 5, Additional file 6).

**Figure 6:**
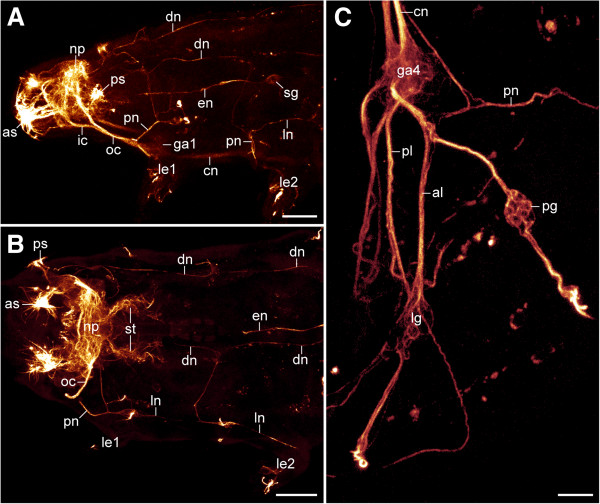
**Organisation of the peripheral nervous system in the tardigrade *Macrobiotus *cf. *harmsworthi. ***Combined anti-tyrosinated and anti-acetylated α-tubulin immunolabelling. Confocal micrographs. **(A)** Anterior body half of a specimen in lateral view (anterior is left). **(B)** Anterior body half of a specimen in dorsolateral view (anterior is left). **(C)** Posterior body region of a specimen in lateral view (anterior is up). Abbreviations: al, anterior leg nerve; as, anterolateral sensory field; cn, connective; dn, dorsolateral nerve tract; en, oesophageal neurite; ga1, first trunk ganglion; ga4, fourth trunk ganglion; ic, inner connective; le1–le2, position of legs 1 and 2; lg, leg ganglion; ln, lateral nerve tract; np, central brain neuropil; oc, outer connective; pg, peripheral ganglion; pl, posterior leg nerve; pn, peripheral nerve; ps, posterolateral sensory field; sg, stomatogastric ganglion; st, neurite bundles innervating the stylet musculature. Scale bars: 20 μm **(A**, **B)** and 5 μm **(C)**.

**Figure 7:**
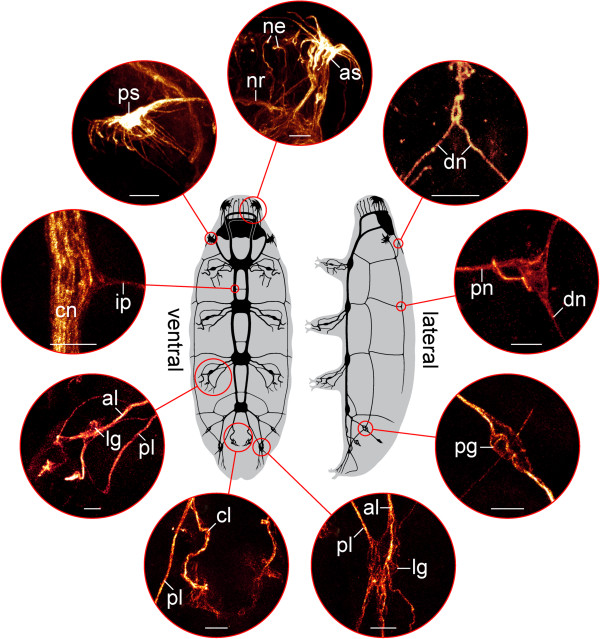
**Organisation of the tardigrade nervous system.** Diagrams in the centre illustrate specimens of the tardigrade *Macrobiotus* cf. *harmsworthi* in ventral and lateral views (anterior is up). Nerve tracts are indicated in black. Circular insets show details (confocal micrographs) of the nervous system based on anti-tyrosinated and anti-acetylated α-tubulin immunolabelling. Abbreviations: al, anterior leg nerve; as, anterolateral sensory field; cl, cloacal nerves; cn, connective; dn, dorsolateral nerve tract; ip, interpedal commissure; lg, leg ganglion; ne, neurites supplying the peribuccal lamellae; nr, nerve ring innervating the peribuccal lamellae; pg, peripheral ganglion; pl, posterior leg nerve; pn, peripheral nerve; ps, posterolateral sensory field. Scale bars: 5 μm.

**Figure 8:**
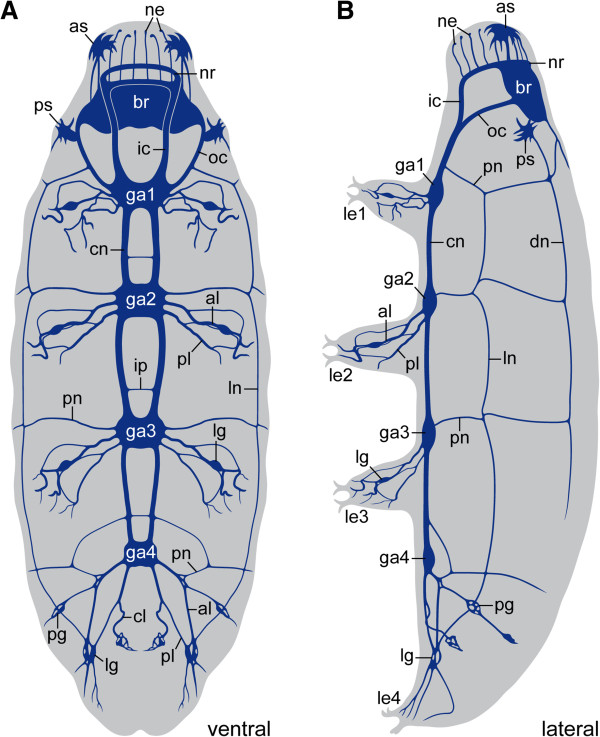
**Diagrams showing the organisation of the tardigrade nervous system.** Reconstructions of nerve tracts are based on anti-tubulin immunolabelling in *Macrobiotus* cf. *harmsworthi*. Anterior is up. **(A)** Ventral view. **(B)** Lateral view. Abbreviations: al, anterior leg nerve; as, anterolateral sensory field; br, brain; cl, cloacal nerves; cn, connective; dn, dorsolateral nerve tract; ga1–ga4, trunk ganglia 1 to 4; ic, inner connective; ip, interpedal commissure; le1–le4, legs 1 to 4; lg, leg ganglion; ln, lateral nerve tract; ne, neurites supplying the peribuccal lamellae; nr, nerve ring innervating the peribuccal lamellae; oc, outer connective; pg, peripheral ganglion; pl, posterior leg nerve; pn, peripheral nerve; ps, posterolateral sensory field.

Each trunk ganglion gives rise to two pairs of leg nerves, one anterior pair and a posterior pair that project postero-laterally into the legs (Figures 4A, B, 5A, 6C, 7 and 8A, B). This peculiar posterior course of the leg nerves is due to an anteriorly shifted position of each trunk ganglion with respect to the corresponding leg pairs (Figure 2B–D and Additional file 2, Additional file 4). Though less evident in the first leg-bearing segment, this shift is clearly present in all trunk segments (Figure 6A and Additional file 4). Each leg nerve gives rise to additional branches and the anterior leg nerve is associated with a peripheral ganglion lying at the basis of each leg (Figures 5A, 6C, 7 and 8A, B). While the branching pattern of the leg nerves is similar in the first three leg pairs, the branches are part of a complex network in the fourth leg-bearing segment (Figures 5A–D, 6C, 7 and 8A, B). Among other tracts, this network contains a pair of nerves embracing the cloaca. These cloacal nerves terminate in two large, serotonin-like and allatostatin-like immunoreactive, platelet-shaped structures devoid of nuclei (Figure 5B, C and Additional file 5). Moreover, they are associated with two RFamide-like immunoreactive cell bodies (arrows in Figure 5D and Additional file 4).

Additional peripheral tracts form an orthogon-like grid encompassing the body and consisting of two pairs of longitudinal and four pairs of transverse tracts linked to each other (Figures 6A–C, 7 and 8A, B). The first pair of transverse peripheral nerves is associated with the outer connectives, whereas the second and the third pairs project laterally from the second and third trunk ganglia. The fourth pair is part of the anastomosing network of peripheral tracts in the fourth leg-bearing segment (Figures 5A, 6C and 8A). The lateral pair of longitudinal tracts interconnects the transverse peripheral nerves on each side of the body, whereas the dorsolateral pair of longitudinal tracts runs further dorsally and is linked to the dorsomedian portion of the brain via a convergent pair of nerve tracts (Figure 7 and 8B).

### Position of leg nerves and localisation of motor neurons in the onychophoran *Euperipatoides rowelli*

**Figure 9:**
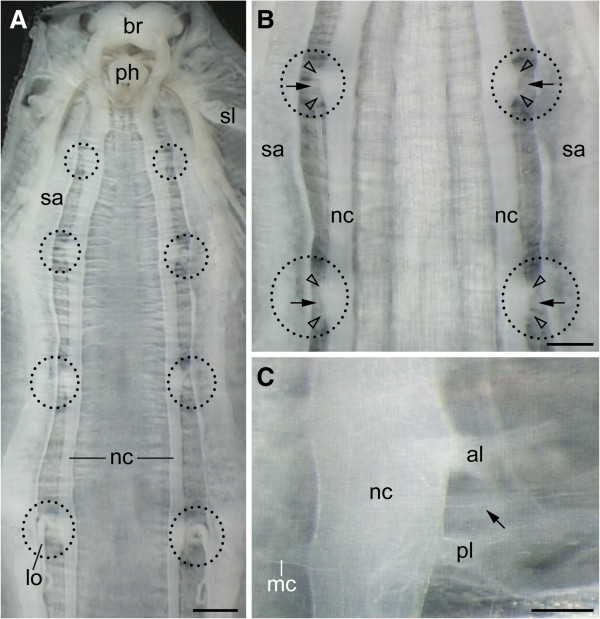
**Organisation of the ventral nervous system and alignment of leg nerves in the onychophoran *Euperipatoides rowelli*.** Light micrographs of a dissected specimen with removed digestive and genital tracts. Anterior is up. Arrows point to nephridial ducts. **(A)** Internal view of ventral body wall showing the position of legs (black dotted circles). **(B)** Detail of leg nerves (arrowheads) extending into each limb. **(C)** Detail of nerve cord showing the position of the anterior and posterior leg nerves. Abbreviations: al, anterior leg nerve; br, brain; lo, labyrinth organ (modified nephridium of the fourth leg-bearing segment); mc, median commissure; nc, nerve cord; ph, remnant of the pharynx; pl, posterior leg nerve; sa, salivary gland; sl, slime gland. Scale bars: 500 μm **(A)**, 300 μm **(B)** and 100 μm **(C)**.

In contrast to the tardigrade nervous system, the nerve cords of the onychophoran *Euperipatoides rowelli* lack segmental ganglia and somata-free connectives (Figure 9A, B). The neuronal cell bodies are instead distributed along the entire nerve cord. Hence, there is no clear border delineating a defined neuromere. The only segmentally repeated structures associated with nerve cords are the paired nerves supplying each leg. As found for the three tardigrade species studied, each leg of the onychophoran *E. rowelli* is also innervated by an anterior and a posterior leg nerve (Figure 9C). However, in contrast to the tardigrade nervous system, the bases of the two leg nerves are not shifted anteriorly but take a direct lateral course into each leg (Figure 9A–C).

**Figure 10:**
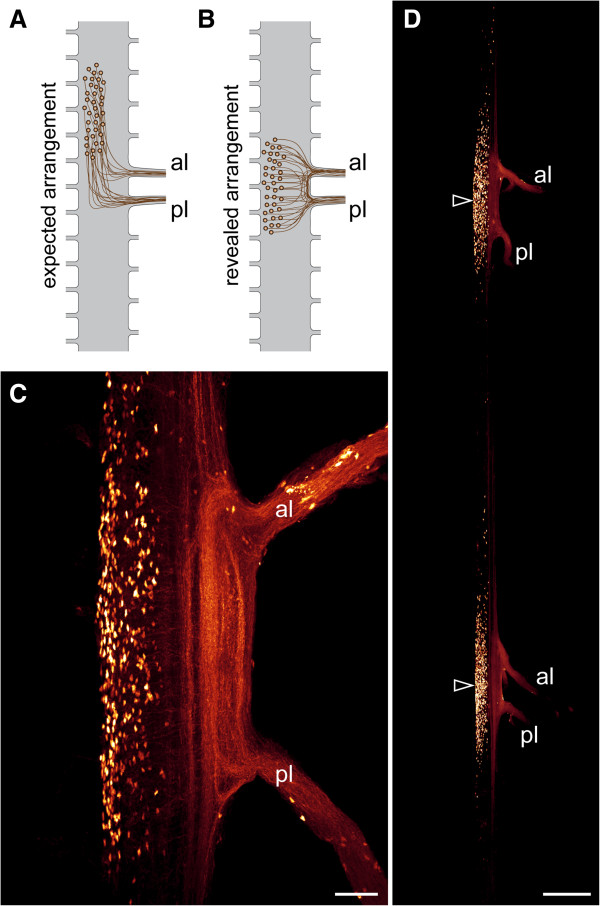
**Retrograde tracing of motor neurons associated with leg nerves in the onychophoran *Euperipatoides rowelli*. (A)** Diagram of expected, anteriorly shifted arrangement of neuronal cell bodies supplying the legs, if parasegments were present. **(B)** Diagram of revealed arrangement. Note that the neuronal cell bodies supplying the legs are not shifted anteriorly but lie next to the root of each leg nerve. **(C, D)** Retrograde fills of leg nerves with dextran coupled to tetramethylrhodamine in dissected nerve cords of the onychophoran *Euperipatoides rowelli*. Confocal micrographs. Note the position of neuronal cell bodies next to the roots of the leg nerves (arrowheads in **D**). Abbreviations: al, anterior leg nerve; pl, posterior leg nerve. Scale bars: 50 μm **(C)** and 250 μm **(D)**.

The cell bodies of motor neurons in *Euperipatoides rowelli* could nonetheless still be shifted anteriorly, as in tardigrades (Figure 10A). To clarify this, we performed retrograde fills of the leg nerves using dextran coupled to tetramethylrhodamine. Our data show that the cell bodies of locomotory neurons innervating the onychophoran legs are grouped around the roots of the two leg nerves (Figure 10B–D). Thus, in contrast to the ventral nervous system of tardigrades, neither the bases of leg nerves nor the cell bodies of motor neurons are shifted anteriorly with respect to each leg pair in onychophorans.

## Discussion

### Interpedal commissures and grid-like arrangement of peripheral nerves: Remnants of an ancestral orthogonal nervous system?

Previous studies have shown that an orthogonal organisation of the nervous system, characterised by a regular grid of longitudinal tracts interconnected by numerous ring commissures, was most likely present in the last common ancestor of panarthropods, as it occurs in various invertebrates, including the onychophorans [8,27,28]. Thus, the ganglionated, “rope ladder-like” nervous system of arthropods might have evolved from such an ancestral orthogonal architecture. This hypothesis receives support from our data, as we observed a regular, grid-like arrangement of longitudinal and transverse peripheral tracts in the tardigrade nervous system. Although the transverse tracts do not form complete rings, together with the paired lateral and dorsolateral nerve tracts, they might be remnants of an ancestral orthogonal arrangement, which might have been present in the last common ancestor of Panarthropoda [27,28].

**Figure 11:**
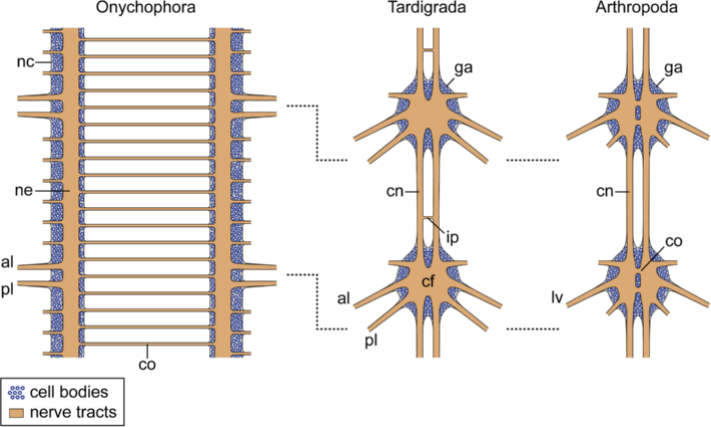
**Schema comparing the ventral nervous systems of Onychophora, Tardigrada and Arthropoda.** Hatched lines indicate the position of legs. Note the anteriorly shifted arrangement of ganglia relative to the legs in tardigrades and arthropods. Diagrams of the onychophoran and arthropod nervous systems according to Mayer and Harzsch [26,27] and Richter et al. [21]. Abbreviations: al, anterior leg nerve; cf, central fibre mass; cn, somata-free connective; co, commissure; ga, ganglion; ip, interpedal commissure; lv, leg nerve; nc, nerve cord; ne, neuropil; pl, posterior leg nerve.

Furthermore, our data revealed additional commissures in the ventral nervous system of the three tardigrade species studied, which lie outside the trunk ganglia, in the interpedal regions, where they link the paired longitudinal connectives with each other (Figure 11). Although these commissures have long been known from tardigrades [29-31], Marcus [22] questioned their neural identity in his monograph, which might have been the reason why these commissures were neglected by most subsequent authors (but see ref. [25]). Nevertheless, our data clearly show that the interpedal commissures belong to the ventral nervous system of tardigrades. These commissures might be remnants of numerous median commissures, which were present in the pedal and interpedal regions in the last common ancestor of Panarthropoda and have persisted in extant onychophorans [8,26-28], whereas they were reduced completely in arthropods (Figure 11).

### Evidence for the homology of segmental ganglia in tardigrades and arthropods

A previous immunocytochemical study [32] has suggested that the organisation of the trunk ganglia in the tardigrade *Macrobiotus hufelandi* is principally different from that in arthropods, on the grounds that no contralateral projections or commissures linking the two hemiganglia were detected. Based on this finding, the contralateral commissures have been reinterpreted as a derived feature (=synapomorphy) of Onychophora and Arthropoda to the exclusion of Tardigrada [33]. However, our immunocytochemical and electron microscopic data clearly show contralateral fibres and bundles of neurites in all four trunk ganglia in specimens of *Macrobiotus* cf. *harmsworthi*, *Paramacrobiotus richtersi* and *Hypsibius dujardini*, thus confirming previous findings from other tardigrade species, including *Macrobiotus hufelandi*[22,23,25,34]. Therefore, contrary to this previous assumption [33], the occurrence of contralateral fibres and commissures in the ventral nervous system does not support the proposed sister group relationship of Onychophora and Arthropoda, as these structures also occur in the tardigrade ganglia and, therefore, were most likely present in the last common ancestor of Panarthropoda.

Likewise, the segmental sets of serotonergic neurons occurring in the ventral ganglia of chelicerates, myriapods, crustaceans and insects [35] have been regarded as a derived feature (=autapomorphy) of Arthropoda [33]. They were unknown in tardigrades, while in onychophorans serotonin-like immunoreactive somata are distributed throughout each nerve cord [26,27]. However, our results show that segmentally repeated sets of serotonin-like immunoreactive (and other) neurons are indeed present in the tardigrade ganglia, suggesting that they are either a synapomorphy of tardigrades and arthropods or an ancestral feature of Panarthropoda rather than an autapomorphy of Arthropoda [33].

Moreover, our data revealed an anteriorly shifted position of trunk ganglia with respect to each leg pair in the three tardigrade species studied, which is also seen in the illustrations of other tardigrade species [22,23,25,29-32,36,37]. Notably, a corresponding anterior shift in position of ganglia occurs in representatives of all major arthropod groups [38], which might be due to the embryonic origin of ganglia. During embryogenesis, the formation of ganglia coincides with parasegments, the initial functional units of the arthropod embryo, which are shifted anteriorly with respect to the adult segments [38,39]. At the end of embryogenesis, the ganglia retain their anteriorly shifted, parasegmental arrangement, whereas the position of other organs, including the legs, corresponds to the adult segments [38]. Consequently, the characteristic, anteriorly shifted position of ganglia can be taken as evidence of parasegments in tardigrades.

This assumption receives support from the expression pattern of the Engrailed protein in the tardigrade embryo, which is localised in the posterior ectoderm of each segment, thus resembling the situation in arthropods [40]. This suggests that the segment polarity gene *engrailed* has a conserved function in establishing the morphological boundaries between segments in both tardigrades and arthropods. In contrast, *engrailed* might play a different role in Onychophora, as the early expression domain of this gene extends beyond the segmental furrow and no precise cellular boundary is evident between *engrailed* and *wingless* domains in the onychophoran embryo [41].

Furthermore, the results of our neuronal tracing experiments using retrograde fills revealed no anterior shift in position of neurons supplying the legs in the onychophoran *Euperipatoides rowelli*, which would be expected, if parasegments were present (cf. diagrams in Figure 10A, B). To our knowledge, there is no indication of a similar anterior shift of neurons in any other group of segmented or “pseudosegmented” protostomes such as the kinorhynchs and annelids, since their ganglia are typically located either midway or even posteriorly within each segment [42-47]. Thus, irrespective of whether the segmental ganglia are homologous across these distantly related protostome taxa, their anteriorly shifted (i.e., parasegmental) position in each segment is unique to tardigrades and arthropods, suggesting that these structures are unlikely to have evolved independently in these two animal groups (Figure 1C, E, G).

Hence, the ventral chain of ganglia in a parasegmental position is either a synapomorphy of Tardigrada and Arthropoda (Figure 1B), or it might have been lost in the onychophoran lineage (Figure 1D, F, H). We favour the first alternative, as any other scenario would require additional evolutionary steps and is therefore less parsimonious. In particular, a modification of the ventral chain of ganglia into a pair of widely separated medullary nerve cords, which occur in onychophorans [26-28], cannot be explained by a simple loss of segmental ganglia. Rather, several modifications, including reversals back to ancestral states, would be required. First, the neuronal cell bodies, which had been accumulated to segmental ganglia, would have to be re-dispersed along each nerve cord (cf. Figure 11). Second, the nerve cords would have to be separated from each other secondarily to take up a ventrolateral position on each side of the body. Third, the median commissures would have to have lost their segmental arrangement and to be multiplied along the body. Fourth, the motor neurons supplying each leg would have to be relocated from an anteriorly shifted (parasegmental) to a position in the middle of each segment. Based on these arguments, we consider an evolutionary scenario, which first proposes the presence of segmental ganglia in onychophorans and then assumes a reversal back to the ancestral orthogonal, medullary organisation of their nervous system, as very unlikely.

## Conclusions

Unfortunately, all too often morphological and embryological data are either dismissed or simply mapped on trees generated by using molecular methods. This is problematic, in particular if one is dealing with a long-branch taxon, such as Tardigrada, the position of which is ambiguous, as evidenced by conflicting topologies published each year [2-4,7,10-13,16]. In this case, a careful evaluation of morphological characters might prove helpful. We have shown here that tardigrades and arthropods share a parasegmental organisation of the nervous system, with anteriorly shifted ganglia relative to each leg pair. The ganglia are linked by paired somata-free connectives along the body and the hemiganglia are joined by contralateral projections crossing the midline. Within the ganglia, segmentally repeated sets of serotonin-like (and other) immunoreactive neurons are found.

These neuroanatomical data speak in favour of the sister group relationship of Tardigrada and Arthropoda [1,8,16], i.e., the monophyly of Tactopoda [1], but contradict recent analyses of molecular data, which propose a sister group relationship of tardigrades either to Onychophora [11], to Nematoda [13], or to Onychophora plus Arthropoda [12]. The monophyly of Tactopoda receives additional support by the occurrence of stomatogastric ganglia associated with the third body segment in both tardigrades and arthropods, which are lacking in other animal groups [24]. Moreover, the segmentation of the longitudinal musculature might also support this relationship, as the longitudinal musculature does not show a segmental arrangement in onychophorans [48] and other ecdysozoans, except for kinorhynchs (a condition, which has been interpreted as derived in these animals [15,49-51]). In our view, therefore, it is premature to regard the position of Tardigrada as the sister group of Onychophora and Arthropoda as “resolved” [12]. On the basis of our data, we instead suggest that the segmental ganglia are a synapomorphy uniting Tardigrada and Arthropoda to the exclusion of Onychophora.

## Methods

### Specimens

Specimens of *Macrobiotus* cf. *harmsworthi* Murray, 1907 (Eutardigrada, Macrobiotidae) were obtained from moss samples collected in the Volkspark Großdeuben near Leipzig (Saxony, Germany; N 51°14’, E 12°23’). Specimens of *Paramacrobiotus richtersi* (Murray, 1911) (Eutardigrada, Macrobiotidae) were obtained from soils of the Berzdorf post-mining sites near Görlitz, Saxony, in April 1999, and cultured thenceforth as described previously [52]. Specimens of *Hypsibius dujardini* Doyère, 1840 (Eutardigrada, Hypsibiidae) were purchased from Sciento (Manchester, United Kingdom) and cultured in Petri dishes filled with commercial mineral water (Volvic). They were fed with algae (*Chlorococcum* sp.) once a month. Specimens of *Euperipatoides rowelli* Reid, 1996 (Onychophora, Peripatopsidae) were obtained from rotted logs in the Tallaganda State Forest (New South Wales, Australia; S 35°26', E 149°33'). The necessary permits for the collection of onychophorans were obtained from the Forestry Commission of New South Wales, Australia (Special Purposes Permit for Research no. XX51212).

### Histochemistry and immunocytochemistry on tardigrades

Immunocytochemistry was carried out as described previously [24]. As general markers of neural structures, we used two different antibodies that both stain α-tubulin, a major component of axonal processes, either separately or in combination to increase the intensity of labelling. One antibody, anti-tyrosinated α-tubulin (Sigma-Aldrich, St. Louis, MO, USA; diluted 1:200), is a monoclonal antibody raised in mice against a synthetic peptide (T13) containing 11 C-terminal amino acids of α-tubulin from porcine brain plus an additional N terminal lysine and a C-terminal tyrosine at the C-terminus [53,54]. The second antibody, anti-acetylated α-tubulin (Sigma-Aldrich; diluted 1:500), is also a mouse monoclonal antibody, but directed against acetylated α-tubulin from the outer arm of the sea urchin *Strongylocentrotus purpuratus*. This antibody recognises an epitope located on the α3 isoform of *Chlamydomonas* axonemal α-tubulin, within four residues of Lys^40^ when this amino acid is acetylated [55].

In addition, we used four antisera that recognise different neuromodulators that are common in invertebrates: (1) Anti-serotonin (NT 102 Eugene Tech Inc., NJ, USA; currently Protos Biotech, NJ, USA; diluted 1:1000) is a polyclonal antiserum raised in rabbits against the biogenic amine serotonin, coupled to *Limulus* haemocyanin. Since we cannot fully exclude that the antiserum may bind to serotonin-related substances, in addition to serotonin, we refer to the observed labelling as “serotonin-like” immunoreactivity. (2) Anti-FMRFamide (Incstar, Stillwater, MN, USA; currently ImmunoStar, Hudson, WI, USA; diluted 1:1000) is a polyclonal antiserum raised in rabbits against the neuropeptide FMRFamide coupled to bovine thyroglobulin. We refer to the labelled structures in our specimens as “RFamide-like” immunoreactivity since the antibody labels a variety of peptides terminating with the sequence RFamide. (3) Anti-allatostatin, a polyclonal antiserum directed against the peptide allatostatin I (Dip-allatostatin I, APSGAQRLYGFGL-amide) from the cockroach *Diploptera punctata*, originated from the producer Dr Hans Agricola (Friedrich Schiller University, Jena, Germany). We used it at a dilution of 1:5000. This serum recognises allatostatins I–V, but is almost two orders of magnitude more sensitive to AST-1 than to the other ASTs [56]. The antiserum has been used in cockroaches and locusts [56]. We refer to the labelled structures in our specimens as “allatostatin-like” immunoreactivity. (4) Anti-perisulphakinin, an antiserum against pea-sulfakinin (perisulfakinin), originated from the producer Dr Hans Agricola (Friedrich Schiller University, Jena, Germany), who has used it successfully in various insects as well as in spiders, crustaceans, centipedes and annelids [57-59]. We used it at a dilution of 1:5000. Perisulfakinin belongs to a family of peptides with identical C-terminal sequences (sulphakinis) that share structural similarities with vertebrate cholecystokinin/gastrin peptide and invertebrate FMRFamide-like peptides [57,58]. We refer to the labelled structures in our specimens as “perisulfakinin-like” immunoreactivity.

All antibodies were diluted in 1% normal goat serum in 0.1 M PBS, pH 7.4, containing 1% Triton-X100. Bound antisera were detected using Alexa 488- or 568-tagged secondary antibodies (Invitrogen, Carlsbad, CA, USA) diluted 1:500. After several rinses in PBS, some specimens were incubated for one hour in a solution containing phalloidin-rhodamine (Invitrogen) to stain f-actin as described previously [8,24,60]. After additional rinses in PBS, the DNA-selective fluorescent dyes Hoechst (Bisbenzimide, H33258; Sigma-Aldrich; 1 μg/ml in PBS), SYBR® Green (Invitrogen; 1:10000 in PBS) or RedDot^TM^2 (Biotium, Hayward, CA, USA; 1:250 in PBS) were applied according to the manufacturers’ protocols. Specimens were then rinsed in PBS and mounted between two cover slips in Vectashield mounting medium (Vector Laboratories Inc., Burlingame, CA, USA) or dehydrated in an ethanol series and mounted in methyl salicylate.

### Retrograde staining of onychophoran motor nerves

For neuronal tracing, the onychophoran nerve cords were dissected in physiological saline [61]. Retrograde fills of leg nerves and ring commissures were carried out with dextran coupled to tetramethylrhodamine as described previously [62,63]. Stained nerve cords were dehydrated through a methanol series and mounted between two cover slips in methyl salicylate.

### Transmission electron microscopy on tardigrades

For ultrastructural studies, specimens of the tardigrade *Macrobiotus* cf. *harmsworthi* were prepared using standard methods as described previously [64] and embedded in Epoxy Embedding Medium Kit (Sigma, St. Louis, MO). Ultra-thin sections were cut on a Leica Ultracut UCT25 ultramicrotome (Leica Microsystems, Wetzlar, Germany), stained with uranyl acetate and lead citrate and examined using a Hitachi H500 transmission electron microscope (Hitachi Ltd, Tokyo, Japan) at 75 kV.

### Confocal microscopy, light microscopy and image processing

Specimens were analysed with the confocal laser-scanning microscopes Zeiss LSM 510 META (Carl Zeiss MicroImaging GmbH, Jena, Germany) and Leica TCS STED (Leica Microsystems, Wetzlar, Germany). Confocal image stacks were processed with Zeiss LSM IMAGE BROWSER v4.0.0.241 (Carl Zeiss MicroImaging GmbH) and Leica AS AF v2.3.5 (Leica Microsystems). The overall structure of the onychophoran nervous system and the position of leg nerves were analysed with a stereomicroscope (Leica Wild M10) equipped with a colour digital camera (PCO AG SensiCam, Kelheim, Germany). The overall anatomy of tardigrades was analysed under a transmitted light microscope (Leica Leitz DMR; Leica Microsystems), equipped with a colour digital camera (PCO AG SensiCam). Several micrographs were taken from each specimen at different focal planes and merged to a single projection using the Auto-Blend Layers function in Adobe (San Jose, CA, USA) Photoshop CS4. Final panels and diagrams were produced using Adobe Illustrator CS4 and exported to Tagged Image File Format files.

### Animal ethics

The experiments in this study did not require an approval by an ethical committee. All procedures in this investigation complied with international and institutional guidelines, including the guidelines for animal welfare as laid down by the German Research Foundation (DFG).

## Competing interests

The authors declare that they have no competing interests.

## Authors’ contributions

GM conceived and designed the experiments, analysed the data and wrote the first draft of the manuscript. CM, JR, SK and IP performed the experiments. CM, JR, SK, PAS, IP, KH, ROS, HJP and MS participated in data analysis and helped to draft the manuscript. All authors read, made comments on and approved the final manuscript.

## Supplementary Material

Additional file 1 **Organisation of the brain and the four trunk ganglia in two specimens of the tardigrade *Macrobiotus *cf. *harmsworthi*.** Anti-RFamide-like immunoreactivity. Confocal micrographs of brains in dorsal view and trunk ganglia in ventral view; anterior is up in all images. Note the similar organisation of the brain and trunk ganglia in the two specimens. Arrowheads point to the repeated somata of RFamide-like immunoreactive neurons within the second and third trunk ganglia. Abbreviations: cn, connective; ex, extra-cerebral neuronal cell body; ic, inner connective; np, central brain neuropil; so, neuronal somata. Scale bars: 5 μm. Click here for file

Additional file 2 **Organisation of the ventral nervous system in the tardigrade *Hypsibius dujardini*.** Note the extra-ganglionic, interpedal commissures in front of the second, third and fourth trunk ganglia (arrowheads). Combined anti-tyrosinated and anti-acetylated α-tubulin immunolabelling. Confocal micrographs; anterior is up. (A) Specimen in lateral view. (B) Specimen in ventral view. Abbreviations: al, anterior leg nerve; cn, connective; ga2–ga4, trunk ganglia 2 to 4; lg, leg ganglion; pl, posterior leg nerve; pn, peripheral nerve. Scale bars: 10 μm. Click here for file

Additional file 3 **Organisation of the ventral nervous system in the tardigrade *Macrobiotus *cf. *harmsworthi*.** Anti-RFamide-like immunoreactivity; confocal micrographs; anterior is up in all images. Note the lack of signal in the interpedal commissures (asterisks). Arrowheads (in A and B) point to the repeated somata of RFamide-like immunoreactive neurons within the second and third trunk ganglia. (A) Detail of the first and second trunk ganglia. (B) Detail of the third trunk ganglion. (C) Detail of the fourth trunk ganglion. Abbreviations: cn, connective; ga1–ga4, trunk ganglia 1 to 4. Scale bars: 5 μm. Click here for file

Additional file 4 **Organisation of the ventral nervous system in the tardigrade *Macrobiotus *cf. *harmsworthi.*** RFamide-like immunoreactivity (glow mode) and DNA labelling (light blue); confocal micrographs; anterior is up in all images. (A) Entire specimen in ventral view. Asterisks indicate the position of the interpedal commissures. (B–D) Details of the posterior end showing the position of two RFamide-like immunoreactive somata associated with cloacal nerves (arrows). Abbreviations: cl, cloacal nerve; cn, connectives; ga1–ga4, trunk ganglia 1 to 4; ic, inner connective; le1–le4, walking legs 1 to 4; pg, peripheral ganglion; pn, peripheral nerve. Scale bars: 20 μm (A) and 10 μm (B–D). Click here for file

Additional file 5 **Organisation of the ventral nervous system at the posterior end in the tardigrade *Macrobiotus *cf. *harmsworthi.*** Serotonin-like immunoreactivity (glow mode) and DNA labelling (light blue); confocal micrographs. Arrowheads point to the somata of serotonin-like immunoreactive neurons. (A) Specimen in ventral view; anterior is up. Note the absence of signal in the interpedal commissure (asterisk). (B–D) Details of the posterior end showing the position of two platelet-shaped, serotonin-like immunoreactive structures (=putative release cites) associated with cloacal nerves (arrows). Abbreviations: cl, cloacal nerve; cn, connectives; ga3–ga4, trunk ganglia 3 to 4. Scale bars: 10 μm (A–D). Click here for file

Additional file 6 **Anti-allatostatin immunolabelling in the tardigrade *Hypsibius dujardini. ***Confocal micrographs; anterior is up in all images. Note the lack of signal in the interpedal commissures (asterisks; cf. Additional file 2). Arrowheads (in C) indicated the position of the two platelet-shaped structures associated with cloacal nerves. (A) Details of the brain in dorsal view. (B) Detail of the first and second trunk ganglia in ventral view. (C) Detail of the third and fourth trunk ganglia. Abbreviations: cn, connective; ga1–ga4, trunk ganglia 1 to 4; ic, inner connective; so, somata of allatostatin-like immunoreactive neurons. Scale bars: 10 μm. Click here for file
