# Supplementary figures and images for: Selective neuronal staining in tardigrades and onychophorans provides insights into the evolution of segmental ganglia in panarthropods
Source: BMC Evol Biol. 2013 Oct 24;13:230. doi: 10.1186/1471-2148-13-230 (PMC4015553; doi:10.1186/1471-2148-13-230)

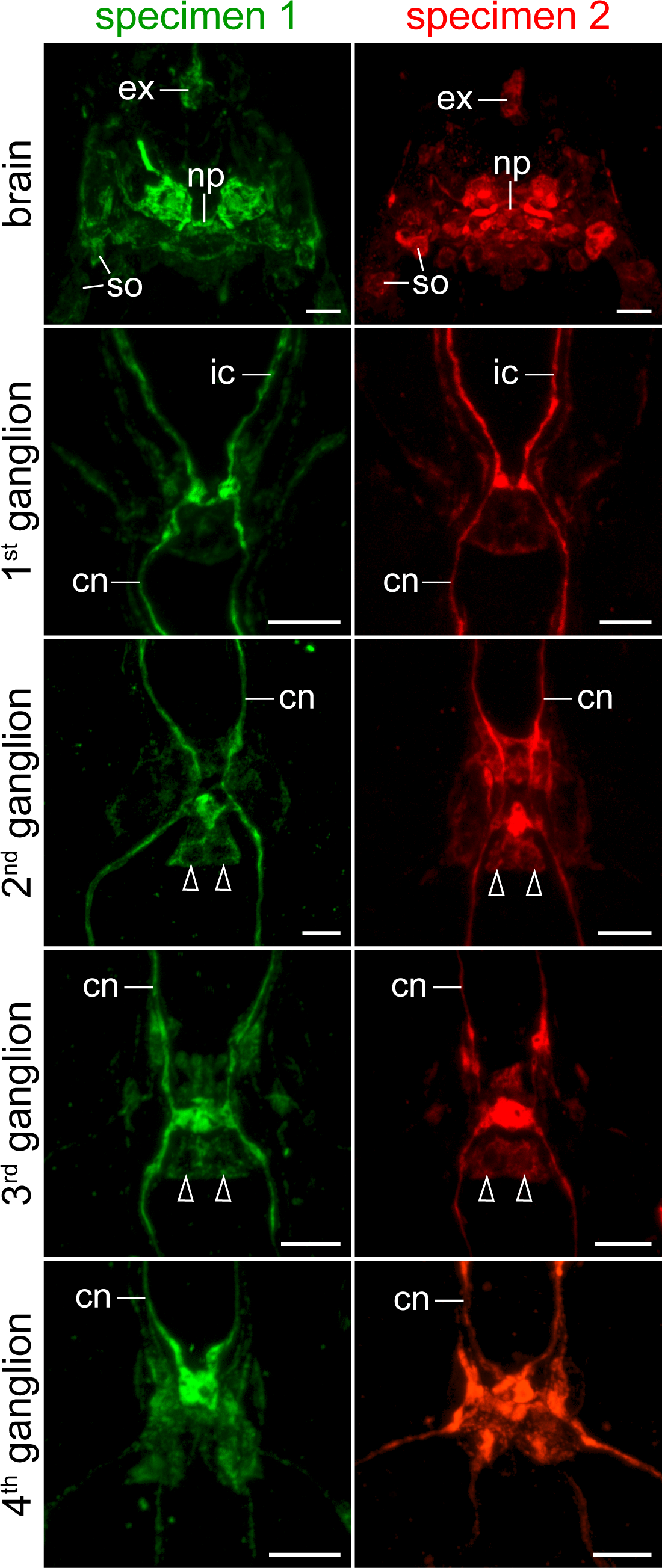

Supplement: Additional file 1 — Organisation of the brain and the four trunk ganglia in two specimens of the tardigrade Macrobiotus cf. harmsworthi. Anti-RFamide-like immunoreactivity. Confocal micrographs of brains in dorsal view and trunk ganglia in ventral view; anterior is up in all images. Note the similar organisation of the brain and trunk ganglia in the two specimens. Arrowheads point to the repeated somata of RFamide-like immunoreactive neurons within the second and third trunk ganglia. Abbreviations: cn, connective; ex, extra-cerebral neuronal cell body; ic, inner connective; np, central brain neuropil; so, neuronal somata. Scale bars: 5 μm. [file 1471-2148-13-230-S1.tiff]

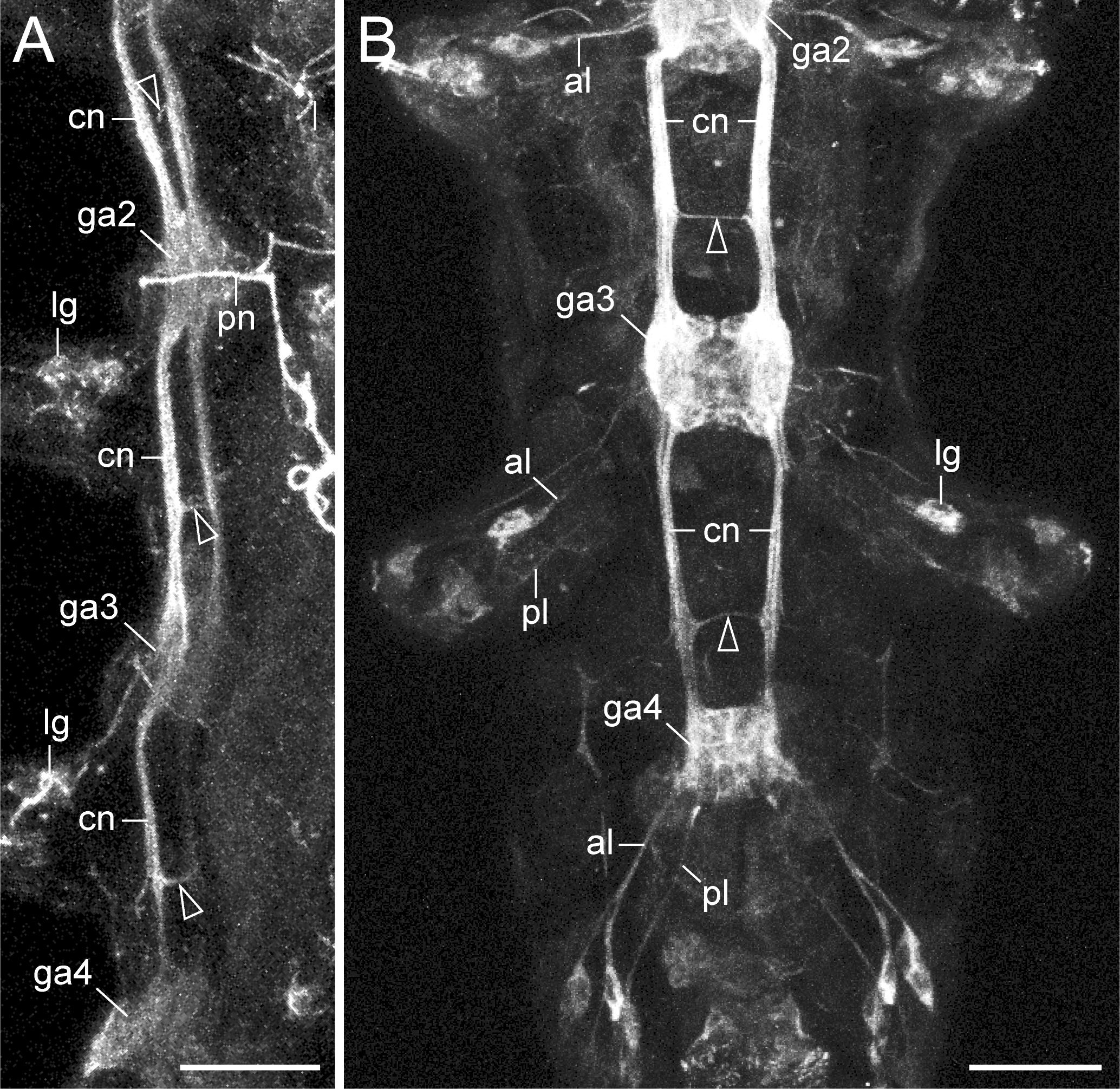

Supplement: Additional file 2 — Organisation of the ventral nervous system in the tardigrade Hypsibius dujardini. Note the extra-ganglionic, interpedal commissures in front of the second, third and fourth trunk ganglia (arrowheads). Combined anti-tyrosinated and anti-acetylated α-tubulin immunolabelling. Confocal micrographs; anterior is up. (A) Specimen in lateral view. (B) Specimen in ventral view. Abbreviations: al, anterior leg nerve; cn, connective; ga2–ga4, trunk ganglia 2 to 4; lg, leg ganglion; pl, posterior leg nerve; pn, peripheral nerve. Scale bars: 10 μm. [file 1471-2148-13-230-S2.tiff]

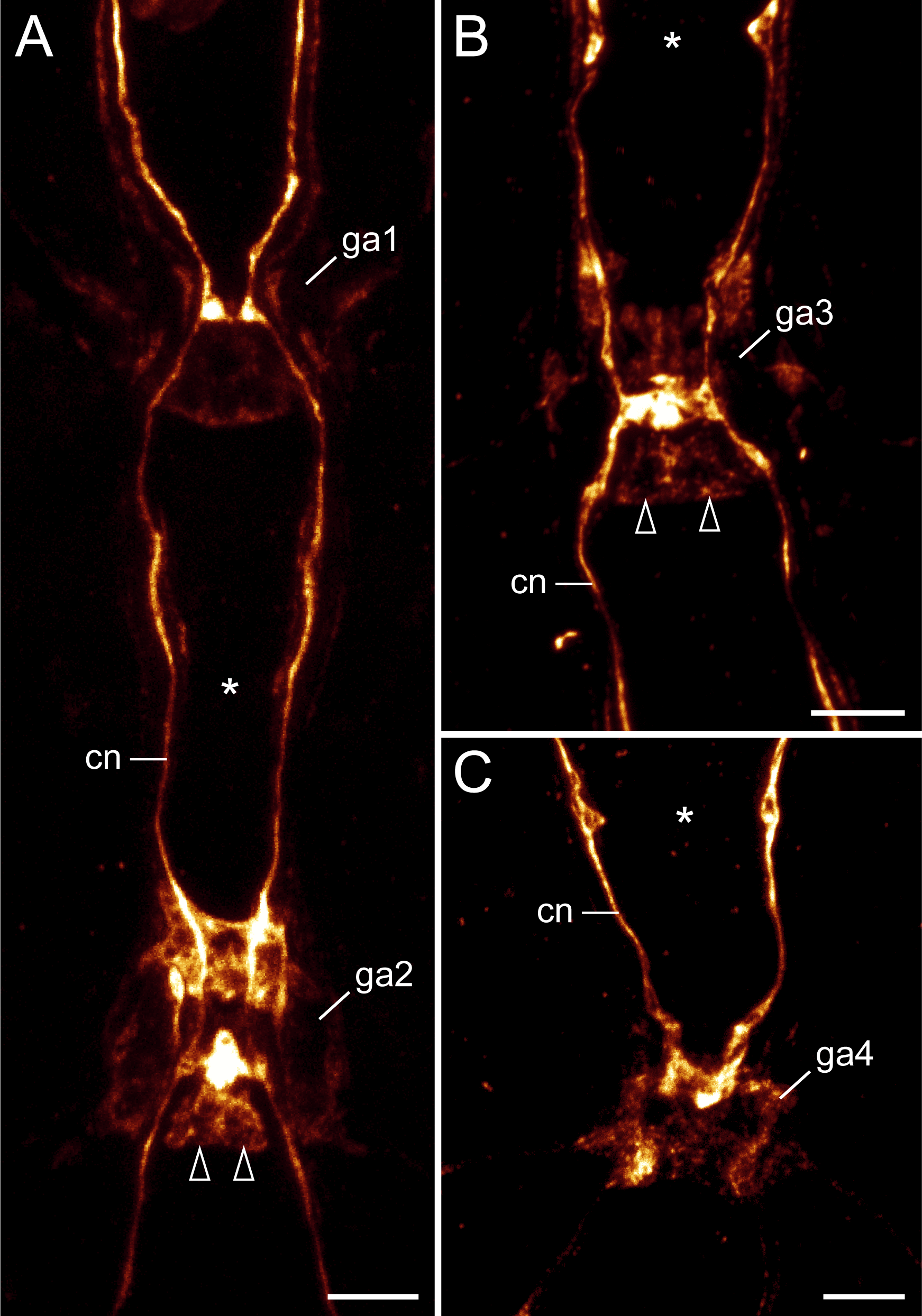

Supplement: Additional file 3 — Organisation of the ventral nervous system in the tardigrade Macrobiotus cf. harmsworthi. Anti-RFamide-like immunoreactivity; confocal micrographs; anterior is up in all images. Note the lack of signal in the interpedal commissures (asterisks). Arrowheads (in A and B) point to the repeated somata of RFamide-like immunoreactive neurons within the second and third trunk ganglia. (A) Detail of the first and second trunk ganglia. (B) Detail of the third trunk ganglion. (C) Detail of the fourth trunk ganglion. Abbreviations: cn, connective; ga1–ga4, trunk ganglia 1 to 4. Scale bars: 5 μm. [file 1471-2148-13-230-S3.tiff]

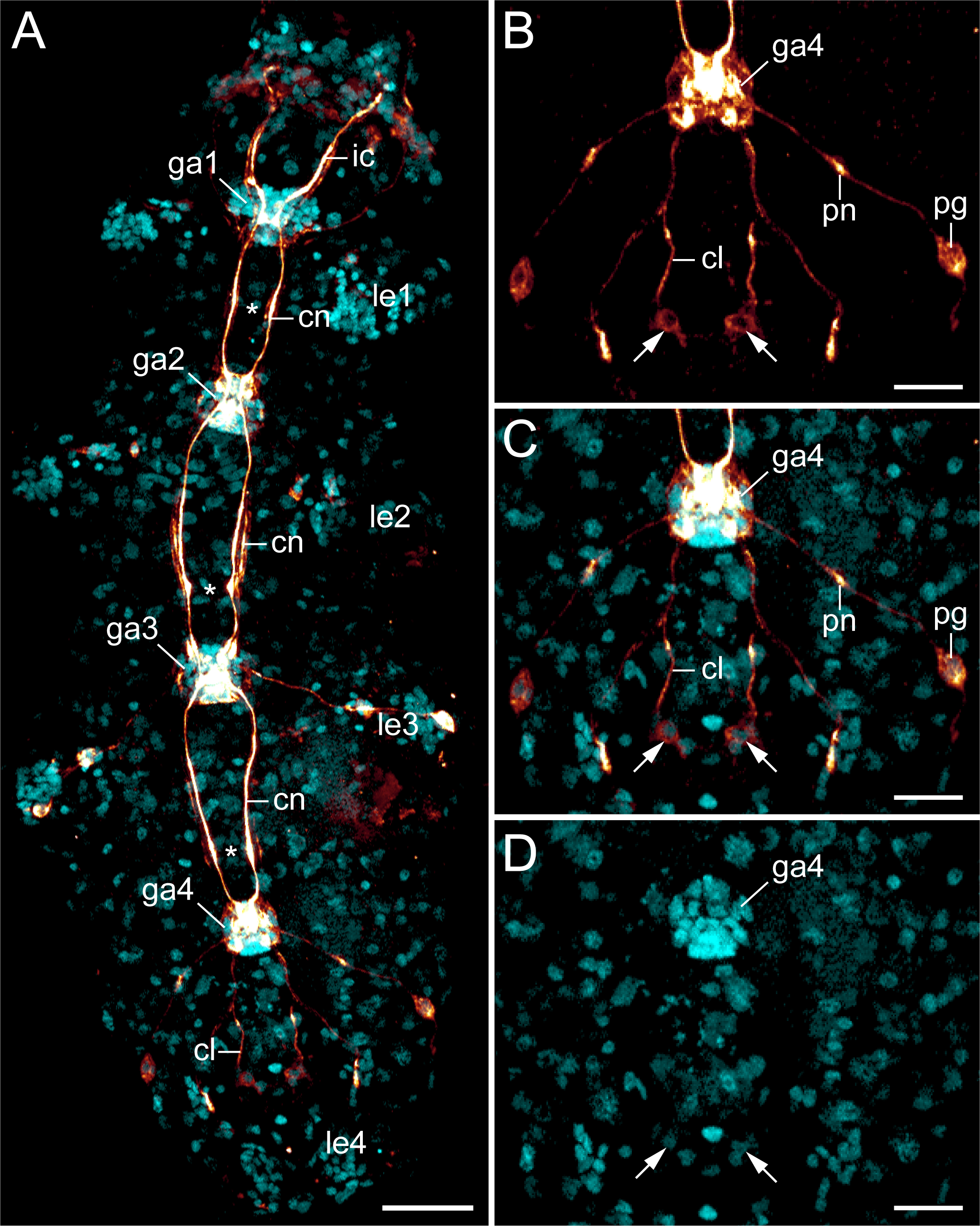

Supplement: Additional file 4 — Organisation of the ventral nervous system in the tardigrade Macrobiotus cf. harmsworthi. RFamide-like immunoreactivity (glow mode) and DNA labelling (light blue); confocal micrographs; anterior is up in all images. (A) Entire specimen in ventral view. Asterisks indicate the position of the interpedal commissures. (B–D) Details of the posterior end showing the position of two RFamide-like immunoreactive somata associated with cloacal nerves (arrows). Abbreviations: cl, cloacal nerve; cn, connectives; ga1–ga4, trunk ganglia 1 to 4; ic, inner connective; le1–le4, walking legs 1 to 4; pg, peripheral ganglion; pn, peripheral nerve. Scale bars: 20 μm (A) and 10 μm (B–D). [file 1471-2148-13-230-S4.tiff]

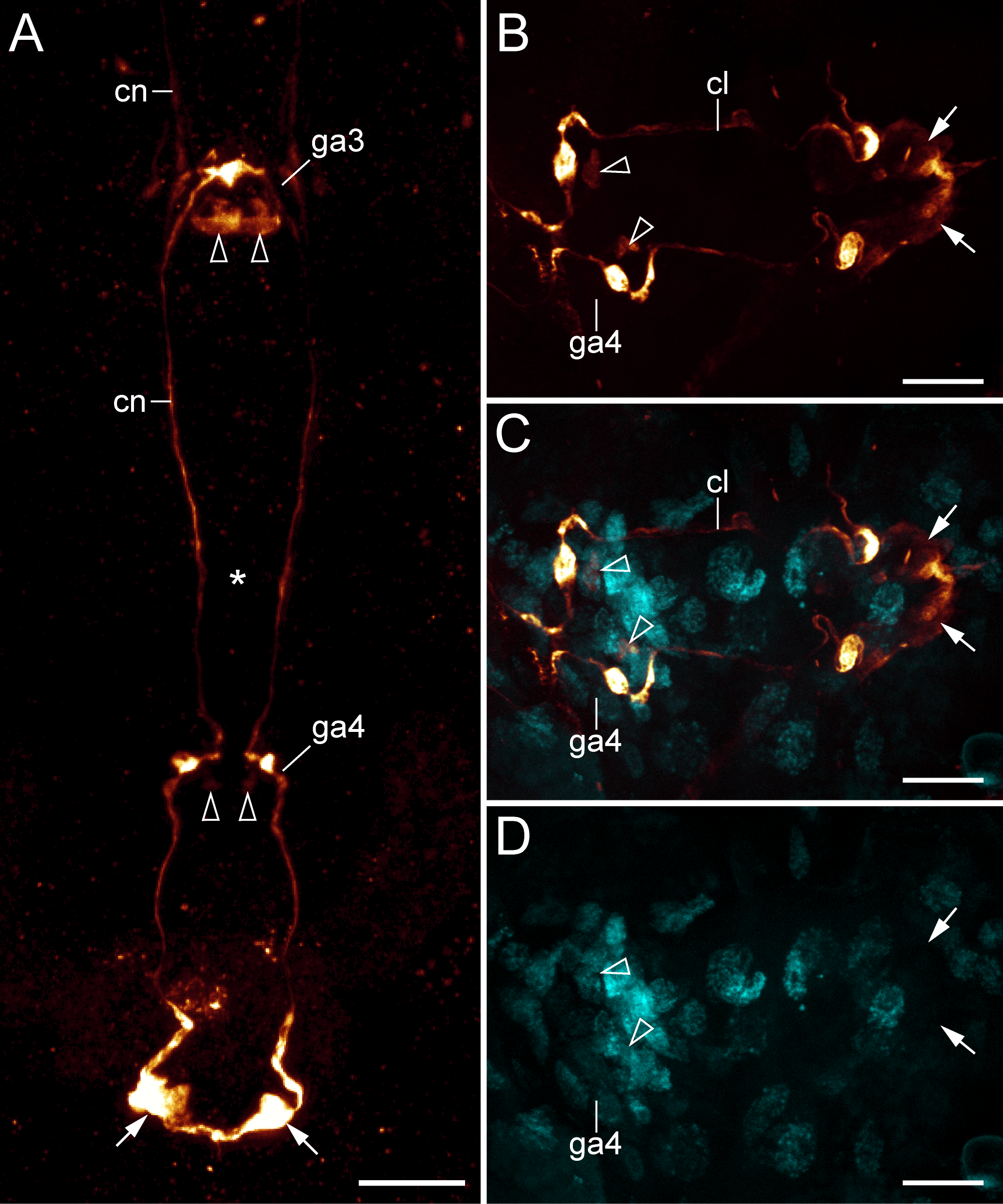

Supplement: Additional file 5 — Organisation of the ventral nervous system at the posterior end in the tardigrade Macrobiotus cf. harmsworthi. Serotonin-like immunoreactivity (glow mode) and DNA labelling (light blue); confocal micrographs. Arrowheads point to the somata of serotonin-like immunoreactive neurons. (A) Specimen in ventral view; anterior is up. Note the absence of signal in the interpedal commissure (asterisk). (B–D) Details of the posterior end showing the position of two platelet-shaped, serotonin-like immunoreactive structures (=putative release cites) associated with cloacal nerves (arrows). Abbreviations: cl, cloacal nerve; cn, connectives; ga3–ga4, trunk ganglia 3 to 4. Scale bars: 10 μm (A–D). [file 1471-2148-13-230-S5.tiff]

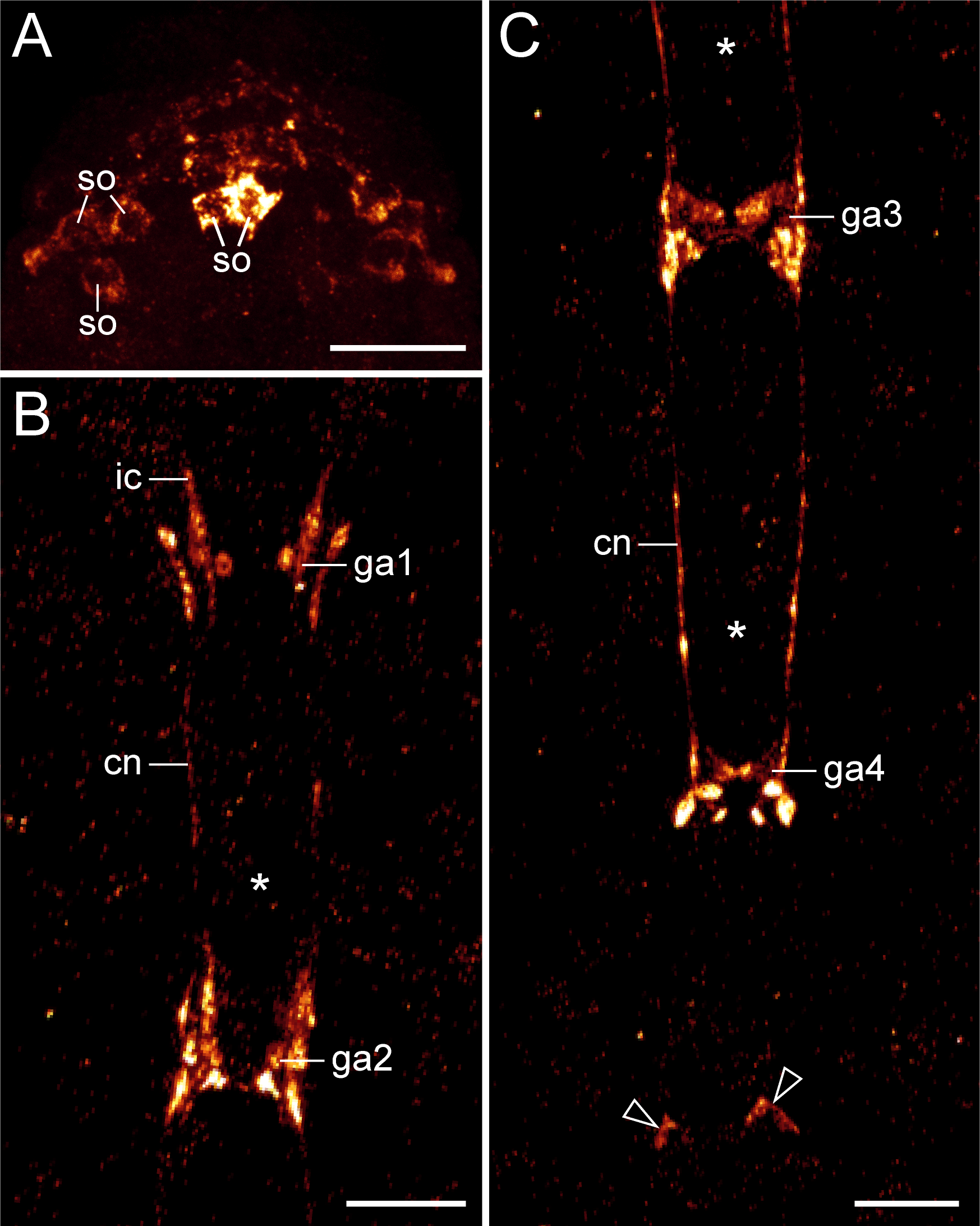

Supplement: Additional file 6 — Anti-allatostatin immunolabelling in the tardigrade Hypsibius dujardini. Confocal micrographs; anterior is up in all images. Note the lack of signal in the interpedal commissures (asterisks; cf. Additional file 2). Arrowheads (in C) indicated the position of the two platelet-shaped structures associated with cloacal nerves. (A) Details of the brain in dorsal view. (B) Detail of the first and second trunk ganglia in ventral view. (C) Detail of the third and fourth trunk ganglia. Abbreviations: cn, connective; ga1–ga4, trunk ganglia 1 to 4; ic, inner connective; so, somata of allatostatin-like immunoreactive neurons. Scale bars: 10 μm. [file 1471-2148-13-230-S6.tiff]
